# Assessment of Knowledge and Attitudes Toward Palliative Care and End-of-Life Decision-Making in Saudi Arabia: A Cross-Sectional Study

**DOI:** 10.7759/cureus.45781

**Published:** 2023-09-22

**Authors:** Fahad N Alsolami, Ibrahim M Alharbi, Jabr N Alsulami, Noura S Albohassan, Layan S Alfraidi, Fares A Alfares, Sherefah I Alsayafi, Mahmoud I Abu Hajar, Tamara Y Alsheikh, Farah M Asad

**Affiliations:** 1 General Practice, Dar Al Uloom University, Riyadh, SAU; 2 General Practice, Alfaisal University, Riyadh, SAU; 3 Public Health, Primary Health Care Center, Hofuf, SAU

**Keywords:** end-of-life ethics, religion and culture, saudi arabia, hospice care, palliative care

## Abstract

Background: Palliative care in Saudi Arabia has witnessed significant recent progress through the establishment of the Saudi Society for Palliative Care and the National Palliative Care Program. The objective of this study was to assess knowledge and attitudes regarding palliative care and end-of-life decision-making in Saudi Arabia's Eastern and Central provinces among individuals residing in these regions.

Methods: Utilizing a cross-sectional survey-based research design, we assessed knowledge and attitudes regarding palliative care and end-of-life decision-making in Saudi Arabia's Eastern and Central provinces. Participants were recruited through purposive sampling via social media. Data collection included demographic information, palliative care knowledge, attitudes toward palliative care, and cultural influences on end-of-life decisions.

Results: A total of 710 participants completed the survey, resulting in a response rate of 85%, with a balanced gender distribution, predominantly aged 25-54. Over half were healthcare providers, many possessing more than 15 years of healthcare experience. A substantial proportion had received formal palliative care training and had personal involvement in end-of-life decisions. While most participants demonstrated a good understanding of palliative care, knowledge gaps, especially regarding its timing, persisted. Generally, participants felt at ease discussing end-of-life care and believed in palliative care's effectiveness. Cultural influences on end-of-life decisions were perceived both positively and negatively, with some facing cultural challenges in palliative care.

Conclusions: This study underscores a promising understanding of palliative care in Saudi Arabia alongside persistent misconceptions. It highlights the necessity for targeted education to rectify misperceptions, particularly concerning the initiation timing of palliative care. Cultural factors strongly impact end-of-life decisions, emphasizing the need for culturally sensitive healthcare discussions and provider training.

## Introduction

Palliative care in Saudi Arabia has witnessed substantial growth and development in recent years, signifying a progressive shift in healthcare priorities. The establishment of the Saudi Society for Palliative Care in 2010 and the initiation of the National Palliative Care Program by the Ministry of Health in 2016 have played pivotal roles in elevating the quality of palliative care services within the country [1]. Palliative care, in this context, aims to enhance the quality of life for individuals grappling with serious illnesses and their families, offering relief from symptoms, pain, and emotional distress, often delivered alongside curative treatments.

The provision of palliative care services has diversified and expanded across Saudi Arabia, encompassing various healthcare settings, including hospitals, primary healthcare centers, and home care [2,3]. Private and non-profit organizations also contribute significantly to the palliative care landscape. Nevertheless, variations in service availability, facility resources, and access to specialized healthcare professionals persist across different regions of the kingdom [4].

The intricate interplay of cultural and religious beliefs, particularly within the framework of Islam, strongly influences attitudes toward palliative care and end-of-life decisions in Saudi Arabia [5]. Islamic principles underscore the sanctity of life and the divine determination of life and death, which can lead to hesitance in discontinuing life-sustaining treatments, even in the advanced stages of terminal illness [5,6]. It is noteworthy that palliative care aligns with Islamic principles by prioritizing the enhancement of life quality and the relief of suffering without hastening death [6].

Despite the increasing acceptance and development of palliative care in Saudi Arabia, significant knowledge gaps persist, necessitating further exploration. These gaps include limited research and the influence of cultural variations, which encompass a diverse range of cultural and regional differences within the country [4,7]. Addressing these gaps assumes critical importance in enhancing the quality of palliative care and end-of-life decision-making in Saudi Arabia, ensuring that these services are harmonized with the unique cultural and religious context of the nation.

## Materials and methods

Study design

This study employed a survey-based cross-sectional research design to assess knowledge and attitudes toward palliative care and end-of-life decision-making among participants in Saudi Arabia.

Participants

The target population for this study comprised individuals residing in the Eastern and Central provinces of Saudi Arabia, representing a diverse cross-section of the Saudi Arabian population. Inclusion criteria encompassed individuals aged 18 and above who were residents of the specified provinces, while there were no specific exclusion criteria.

Participants were recruited through a combination of purposive sampling and open online recruitment, utilizing social media platforms, including Facebook, Twitter, and WhatsApp groups, to ensure representation from a diverse cross section of the population. Recruitment messages included invitations to participate in the online survey, accompanied by links to the survey instrument.

Data collection instrument

The survey questionnaire was developed through an extensive review of relevant literature, existing validated scales, and expert consultation. The questionnaire consisted of four main sections: The *Participant Information* section aimed to gather demographic data, including age, gender, profession, years in healthcare, formal palliative care training, personal involvement in end-of-life decisions, exposure to palliative care, and cultural background. In the *Knowledge* section, participants' knowledge of palliative care was assessed using close-ended questions. These questions included inquiries about the definition of palliative care, its aims, timing of introduction, and compatibility with curative treatments. The* Attitudes Toward Palliative Care* section utilized close-ended questions to explore participants' comfort levels in discussing end-of-life care, their beliefs in the effectiveness of palliative care and their perceptions of the importance of palliative care training for healthcare providers. In the *Cultural Influence on End-of-Life Decisions* section, participants' beliefs about the cultural impact on end-of-life decision-making and their personal experiences with cultural challenges in palliative care were assessed using close-ended questions.

Data collection procedure

The survey was administered online using a secure and user-friendly platform, specifically Google Forms, to facilitate participant responses. The survey link was widely disseminated across selected social media groups, with a particular focus on reaching participants in the Eastern and Central provinces of Saudi Arabia.

Data collection occurred over a predefined period, spanning from January 15, 2023 to February 28, 2023, to ensure the recruitment of a representative sample. The participants had the flexibility to complete the survey at their convenience during this timeframe.

Prior to initiating the survey, the participants were presented with an informed consent statement. This statement outlined the study's purpose, voluntary participation, and data usage. The participants provided informed consent by proceeding with the survey.

Data analysis

The data were analyzed using statistical software, specifically IBM SPSS Statistics for Windows version 26 (released 2019, IBM Corp., Armonk, New York, United States). Descriptive statistics, including frequencies and percentages, were utilized to summarize demographic characteristics, knowledge, attitudes, and perceptions of the participants.

Ethical considerations

This study adhered to ethical guidelines by obtaining informed consent from all participants. Participant identities remained confidential, and data were anonymized during analysis. Ethical approval for this study was obtained from the Ministry of Health, ensuring compliance with ethical standards and participant protection. The Institutional Review Board of Al-Ahsa Health Cluster issued approval (approval no. 32-EP-2023).

## Results

Participant information

A total of 710 participants from Saudi Arabia completed the survey, resulting in a response rate of 85%, offering insights into their knowledge and attitudes toward palliative care and end-of-life decision-making. The demographics of the respondents are as follows: The age distribution revealed that the majority of participants fell within the 25-54 age range, accounting for 63.5% of the total respondents. Specifically, 11.5% were aged 18-24, 24.5% were 25-34, 20.0% were 35-44, and 19.9% were 45-54. Moreover, 23.9% were aged 55 or older, with 11.9% in the 55-64 age group and 12.1% aged 65 or older.

In terms of gender distribution, the responses were relatively balanced, with 50.7% identifying as male and 49.3% as female. The professional backgrounds of the participants encompassed a wide range, with 35.2% being healthcare providers, 8.5% educators or researchers, 14.1% patient or family members, and 42.3% indicating other professions.

In addition, 39.6% of the respondents reported personal involvement in end-of-life decisions, while 60.4% did not. Exposure to palliative care varied among the participants, with 45.4% reporting high to very high exposure, 47.7% reporting moderate to low exposure, and 6.9% reporting very low exposure (Table 1).

**Table 1:** Participant demographics (N=710)

Participant Information		Frequency (n)	Percentage (%)
Age	18-24	80	11.3%
	25-34	170	23.9%
	35-44	140	19.7%
	45-54	140	19.7%
	55-64	85	11.9%
	65 or older	95	13.4%
Gender	Male	360	50.7%
	Female	350	49.3%
Profession	Healthcare provider	250	35.2%
	Educator/researcher	60	8.5%
	Patient/family member	100	14.1%
	Others	300	42.3%
Years in Healthcare	Less than 1 year	50	7.0%
	1-5 years	180	25.4%
	6-10 years	120	16.9%
	11-15 years	80	11.3%
	More than 15 years	280	39.4%
Formal palliative care training	Yes	380	53.5%
	No	330	46.5%
Personal involvement in end-of-life decisions	Yes	280	39.4%
	No	430	60.6%
Exposure to palliative care	Very high	130	18.3%
	High	190	26.8%
	Moderate	170	23.9%
	Low	130	18.3%
	Very low	90	12.7%

Palliative care knowledge

When asked about the definition of palliative care, the majority of the respondents (70.3%) correctly identified it as care focused on relieving suffering and improving the quality of life. However, some participants held misconceptions, with 7.9% associating it exclusively with hospice care, 11.1% believing it is provided at the beginning of a serious illness, and 10.7% thinking it only includes pain management.

Concerning the aims of palliative care, the majority understood them correctly, with 88.2% recognizing that it aims to provide emotional support, relieve physical and emotional suffering, and improve the patient's overall quality of life.

The participants' perceptions of when palliative care should be introduced varied, with 61.1% believing it should be introduced as soon as the diagnosis is made. However, some participants had misconceptions, with 10.0% suggesting it should only be introduced in the final weeks of life and 16.3% when the patient requests it. Furthermore, the majority (88.2%) correctly understood that palliative care can be provided alongside curative treatments (Table 2).

**Table 2:** Assessment of palliative care knowledge (N=710)

Palliative care knowledge		Frequency (n)	Percentage (%)
What is palliative care?	Care focused on relieving suffering and improving the quality of life	500	70.4%
	Care provided exclusively in a hospice setting	55	7.7%
	Care provided at the beginning of a serious illness	80	11.3%
	Care that only includes pain management	75	10.6%
Palliative care aims to:	Cure the underlying illness	75	10.6%
	Provide emotional support to patients and families	505	71.1%
	Relieve physical and emotional suffering	465	65.5%
	Improve the patient's overall quality of life	605	85.2%
When should palliative care be introduced?	As soon as the diagnosis is made	435	61.3%
	Only in the final weeks of life	70	9.9%
	When the patient requests it	115	16.2%
	Only when curative treatments have failed	90	12.7%
True or false: Palliative care can be provided alongside curative treatments	True	625	88.0%
	False	85	12.0%

Attitudes toward palliative care

Regarding their comfort level discussing end-of-life care, a substantial proportion of respondents (38.7%) reported feeling very comfortable, while 36.0% felt somewhat comfortable. Only 13.8% expressed some degree of discomfort in discussing end-of-life care.

In terms of belief in the effectiveness of palliative care, 83.5% either strongly agreed or agreed that palliative care can significantly improve the quality of life of patients. Moreover, 88.3% considered palliative care training for healthcare providers to be very important or important (Table 3).

**Table 3:** Attitudes and perceptions regarding palliative care (N=710)

Attitudes toward palliative care		Frequency (n)	Percentage (%)
Comfort discussing end-of-life care	Very comfortable	275	38.7%
	Somewhat comfortable	255	35.9%
	Neutral	85	12.0%
	Somewhat uncomfortable	65	9.2%
	Very uncomfortable	30	4.2%
Belief in palliative care's quality-of-life improvement	Strongly agree	310	43.7%
	Agree	280	39.4%
	Neutral	40	5.6%
	Disagree	60	8.5%
	Strongly disagree	20	2.8%
Importance of palliative care training for healthcare providers	Very important	345	48.6%
	Important	285	40.1%
	Neutral	40	5.6%
	Not very important	25	3.5%
	Not important at all	15	2.2%

Cultural influence on end-of-life decisions

Approximately 52.1% believed that culture had a positive or somewhat positive influence on end-of-life decision-making, while 41.6% perceived no significant influence or a somewhat negative influence. Only 7.0% indicated a strongly negative cultural influence. Regarding encounters with cultural challenges in palliative care, 13.4% reported frequent encounters, 21.8% reported occasional encounters, and 64.8% reported no such encounters (Table 4).

**Table 4:** Cultural factors impacting end-of-life decision-making (N=710)

Cultural influence on end-of-life decisions		Frequency (n)	Percentage (%)
Cultural impact on end-of-life decision-making	Strongly positive influence	155	21.8%
	Somewhat positive influence	215	30.3%
	No significant influence	165	23.2%
	Somewhat negative influence	125	17.6%
	Strongly negative influence	50	7.0%
Encounters with cultural challenges in palliative care	Yes, frequently	95	13.4%
	Yes, occasionally	155	21.8%
	No	460	64.8%

## Discussion

In this study, our primary objective was to evaluate the knowledge and attitudes toward palliative care and end-of-life decision-making among a sample of 710 participants in Saudi Arabia. The insights generated from this research contribute significantly to our understanding of crucial aspects related to palliative care within the Saudi context. It is notable that a substantial majority of participants (70.3%) exhibited a comprehensive understanding of palliative care, correctly identifying it as a form of care that focuses on relieving suffering and enhancing the quality of life. However, it is concerning that some misconceptions persist, with 7.9% associating palliative care exclusively with hospice care and 11.3% believing it should be initiated at the onset of a serious illness.

Despite a generally positive understanding of palliative care, we observed knowledge gaps. For instance, 16.2% of participants believed that palliative care should only be introduced when the patient requests it. This misperception highlights an opportunity for educational interventions that emphasize the importance of early integration of palliative care alongside curative treatments [8-10].

Cultural factors, including the influence of cultural beliefs and values on end-of-life decision-making, were explored in the study. While we did not specifically measure cultural diversity among the survey population, the study aimed to understand participants' beliefs and experiences related to cultural factors as they pertain to palliative care and end-of-life decision-making within the broader Saudi Arabian context. Over half of the participants (52.1%) believed that culture had a positive or somewhat positive influence on these decisions, while 41.6% perceived no significant influence or a somewhat negative influence. These findings highlight the complex interplay between culture, religion, and healthcare decisions. Therefore, it is imperative for healthcare providers to acknowledge these influences and engage in culturally sensitive discussions with patients and their families [11,12].

Understanding participants' comfort levels in discussing end-of-life care is of paramount importance. Nearly 75% expressed some degree of comfort, with 38.7% indicating that they were very comfortable and 36.0% stating that they were somewhat comfortable. However, approximately 18.0% reported varying degrees of discomfort. These findings underscore the critical need for fostering open and empathetic communication in healthcare settings [13].

Approximately 35.2% of the participants reported encountering cultural challenges in palliative care, with 13.4% facing frequent challenges and 21.8% experiencing occasional obstacles. These challenges may encompass differences in treatment preferences, disclosure of diagnoses, and decision-making dynamics within families [14]. Addressing these challenges necessitates culturally sensitive training and guidance for healthcare providers [11,13,15].

The results of this study hold several significant implications for healthcare practice in Saudi Arabia. First, there should be directed efforts to enhance public awareness and understanding of palliative care, emphasizing its holistic approach and advocating for early integration. Second, healthcare providers, particularly those without formal palliative care training, should receive ongoing education to enhance their knowledge and communication skills. Lastly, healthcare institutions should develop policies and practices that respect cultural and religious diversity while ensuring the delivery of high-quality palliative care services.

However, it is important to acknowledge the limitations of this study. Response bias may have influenced the survey-based nature of the research, and the sample may not be entirely representative of the Saudi population due to the reliance on online surveys. In addition, while the study assessed knowledge and attitudes, it did not measure actual behaviors in clinical settings. Future research could explore these aspects in greater depth to provide a more comprehensive understanding of the subject matter.

## Conclusions

This study sheds light on the current landscape of palliative care knowledge and attitudes in Saudi Arabia, revealing both promising understandings and persistent misconceptions. While a significant proportion of participants exhibited a comprehensive grasp of palliative care's essence and benefits, there remains a need for targeted educational initiatives to rectify misperceptions, particularly concerning the timing of palliative care initiation. The study underscores the complex interplay of cultural influences on end-of-life decisions, emphasizing the importance of culturally sensitive healthcare discussions and training for providers. As Saudi Arabia continues to advance its palliative care services, addressing these knowledge gaps and cultural factors will be pivotal in ensuring that palliative care aligns with the unique cultural and religious context of the nation, ultimately enhancing the quality of end-of-life care for individuals and their families.
